# Supplementary material for: Accurate deep neural network inference using computational phase-change memory
Source: Nat Commun. 2020 May 18;11:2473. doi: 10.1038/s41467-020-16108-9 (PMC7235046; doi:10.1038/s41467-020-16108-9)
Supplement: Supplementary file 1 — Supplementary Information [file 41467_2020_16108_MOESM1_ESM.pdf]

Supplementary Information

Accurate deep neural network inference using computational phase-change memory

Vinay Joshi,[1,2] Manuel Le Gallo,[1,a)] Simon Haefeli,[1,3] Irem Boybat,[1,4] S.R. Nandakumar,[1] Christophe Piveteau,[1,3] Martino Dazzi,[1,3] Bipin Rajendran,[2] Abu Sebastian,[1,b)] and Evangelos Eleftheriou[1]

[1)]*IBM Research - Zurich, Säumerstrasse 4, 8803 Rüschlikon, Switzerland*

[2)]*King's College London, Strand, London WC2R 2LS, United Kingdom*

[3)]*ETH Zurich, Rämistrasse 101, 8092 Zurich, Switzerland*

[4)]*Ecole Polytechnique Federale de Lausanne (EPFL), 1015 Lausanne, Switzerland*

(Dated: 30 March 2020)

[a)]Electronic mail: anu@zurich.ibm.com

[b)]Electronic mail: ase@zurich.ibm.com

SUPPLEMENTARY FIGURES

Supplementary Figure 1: ResNet-34 network architecture for ImageNet classification

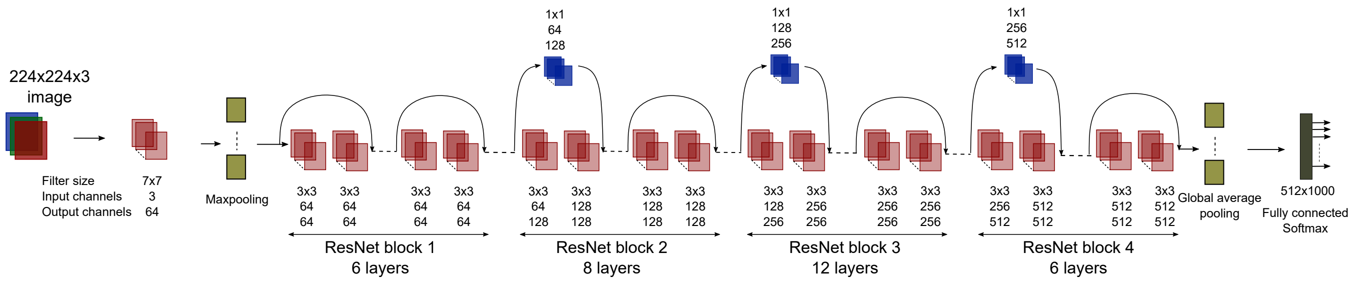

Supplementary Figure 1. **ResNet-34 network architecture for ImageNet classification.**[1] It has 32 convolution layers with 3×3 kernels, 3 convolution layers with 1×1 kernels, a first convolution layer with 7×7 kernels and a final fully-connected layer. The network has 21,797,672 parameters. The first convolution layer downsamples the input by using a stride of 2 pixels, followed by a maxpooling layer with kernel size of 3×3 and stride of 2 to downsample the feature maps to the resolution of 56×56 pixels. Each residual connection with 1×1 convolution and first layer of ResNet blocks 2, 3, 4 downsample the input by using a stride of 2 pixels. A global average pooling layer before the final fully-connected layer downsamples the 7×7 input to 1×1 resolution. The final fully-connected layer computes the output prediction corresponding to 1,000 classes.

Supplementary Figure 2: Impact of different techniques on training with additive noise

a

b

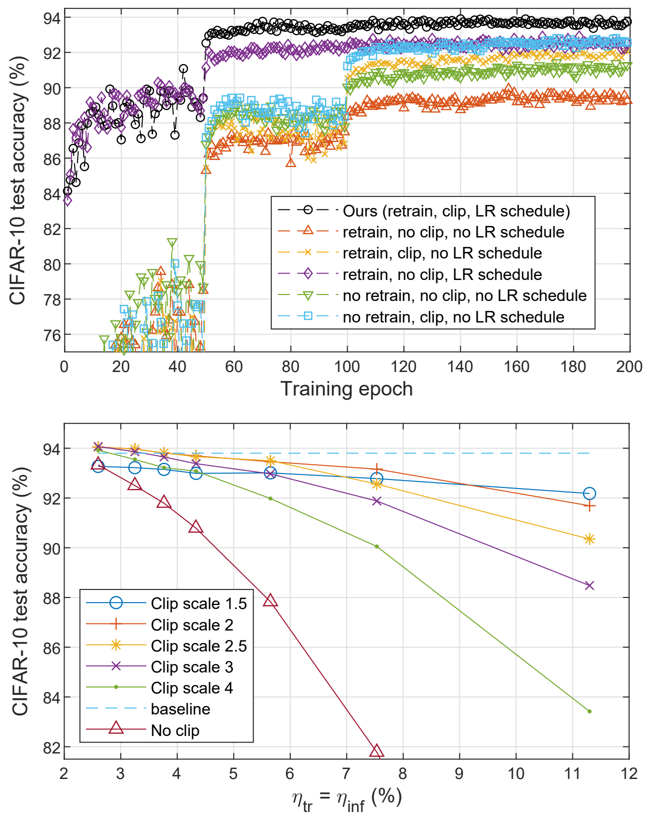

c

Retrain from FP checkpoint	Clip with scale of 2	Use optimal LR scheduling	Best test accuracy	Difference w.r.t Ours
Yes	Yes	Yes	93.9000	0.0000
Yes	Yes	No	92.1800	1.7200
Yes	No	Yes	92.9100	0.9900
Yes	No	No	89.9400	3.9600
No	Yes	No	92.8100	1.0900
No	No	No	91.4300	2.4700

Supplementary Figure 2. **Impact of different techniques on training with additive noise.** **a**, Additive noise training of the ResNet-32 network on CIFAR-10 dataset by using combinations of different training techniques with $\eta_{tr} = \eta_{inf} = 3.8\%$. Our training methodology (in black) that implements all the training techniques, achieves the best performance out of all other possible combinations. **b**, Influence of weight clip scale parameter α on the test accuracy on CIFAR-10 for different amounts of equal training η_{tr} and inference η_{inf} noise. From this experiment we observe that clip scale of 2 is optimal for ResNet-32 network. **c**, Test accuracy improvement from using each of the three training techniques in additive noise training of ResNet-32 network on CIFAR-10 dataset for $\eta_{tr} = \eta_{inf} = 3.8\%$.

Supplementary Figure 3: Impact of injected noise during training on accuracy with PCM

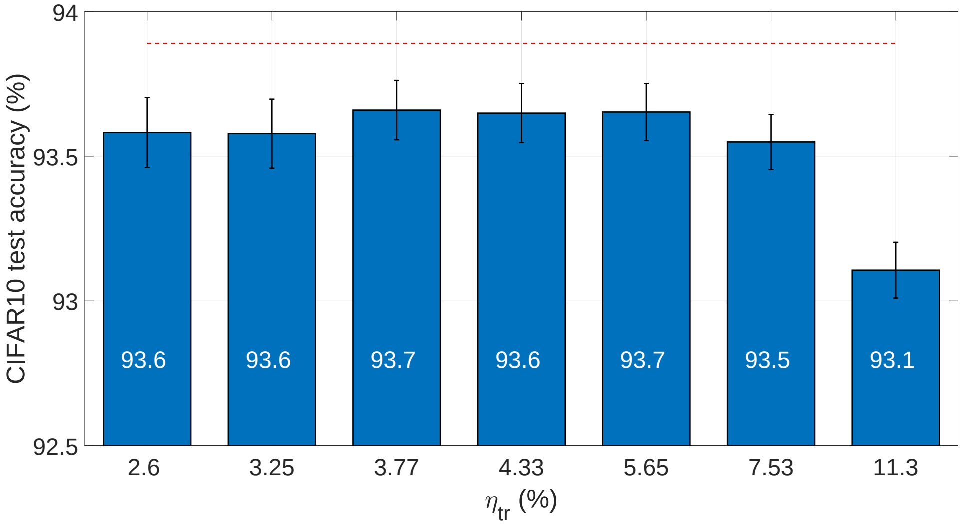

Supplementary Figure 3. **Impact of injected noise during training on accuracy with PCM.** Test accuracy of ResNet-32 on CIFAR-10 after transfer to PCM synapses for different values of relative weight noise η_{tr} used for training. Although a value of $\eta_{\text{tr}} = 3.8\%$ was used in the results presented in the main manuscript, which was determined from hardware characterization, a broad range of values of η_{tr} result in a similar accuracy after transferring the weights to PCM. This shows that only a rough estimate of η_{tr} is necessary to obtain satisfactory results on PCM. The error bars represent the standard deviation over 25 inference runs averaged over 10 training runs.

Supplementary Figure 4: Global drift compensation technique

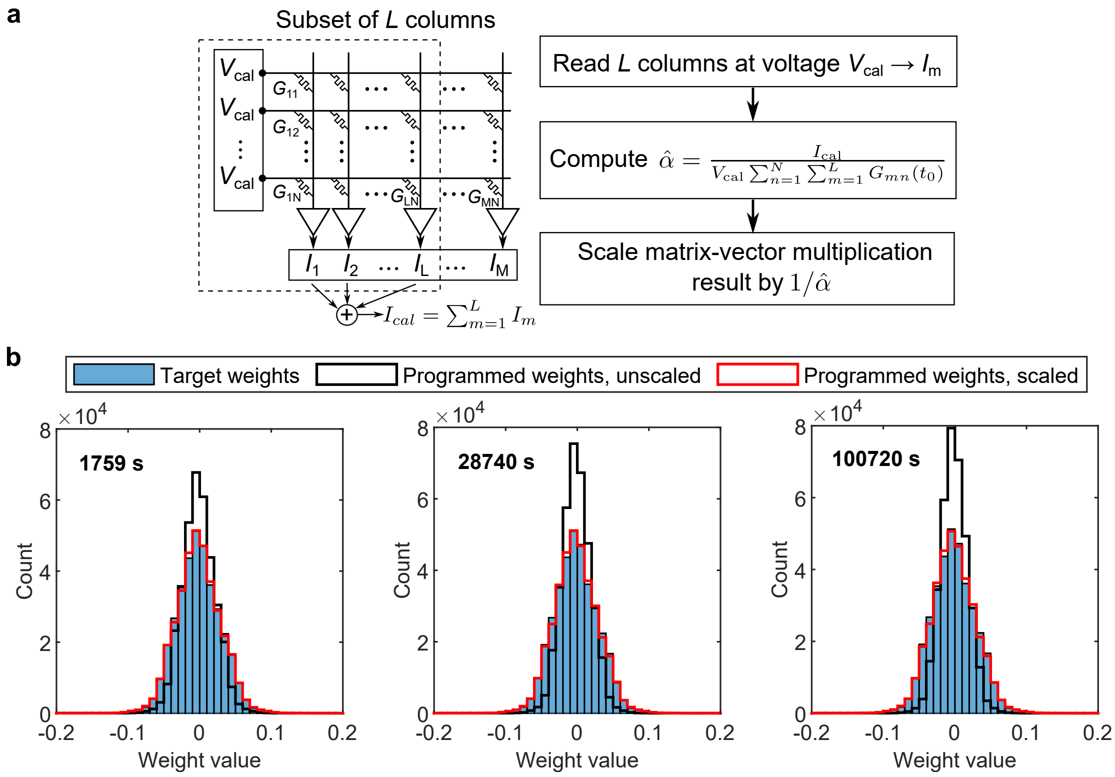

Supplementary Figure 4. **Global drift compensation technique.** **a**, Global drift compensation technique according to Supplementary Ref. 2. **b**, Weight evolution from the PCM chip during the ResNet-32 inference experiment with and without applying the scaling factor obtained from the global drift compensation to the weights. The scaled weights distribution stays much closer to the target weights than the unscaled one.

Supplementary Figure 5: Effect of PCM nonidealities on CIFAR-10 accuracy retention

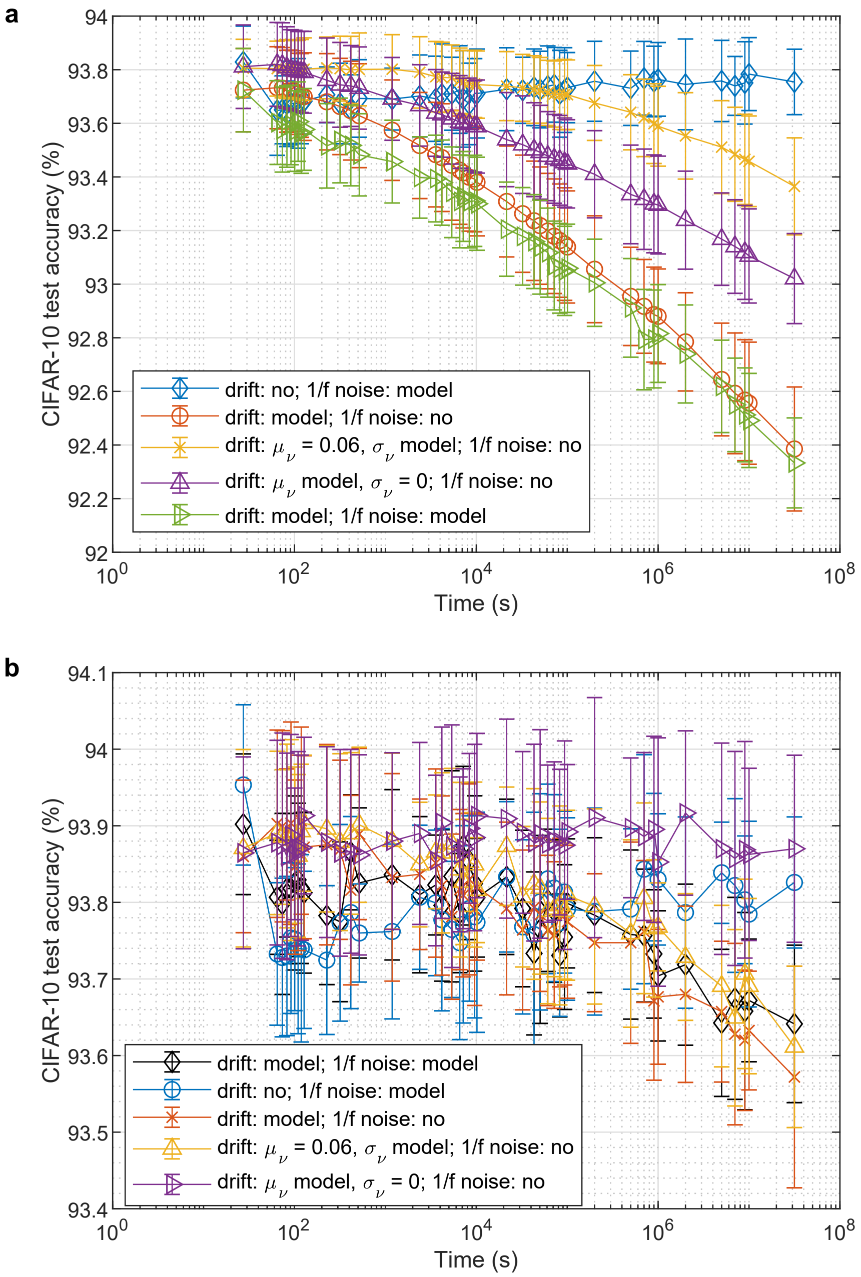

Supplementary Figure 5. **Effect of PCM nonidealities on CIFAR-10 accuracy retention.** Test accuracy of ResNet-32 on CIFAR-10 simulated with different parameters of the PCM model. All those simulations contain the experimentally measured programming noise applied at $T_0 = 27.36$ s (see Supplementary Note 2). In the legend, "model" denotes that the parameters of the PCM model described in Supplementary Note 2 are used for the corresponding nonideality. **a**, Mean accuracy with GDC over 25 inference runs, the error bars corresponding to one standard deviation. The results demonstrate that $1/f$ noise is mainly responsible for the random accuracy fluctuations over time. Drift variability and its dependence on the target conductance are responsible for the monotonous accuracy decrease over time. It can be also seen that the dependence of the mean drift exponent μ_ν on the target conductance state has a more detrimental effect on accuracy than random drift variability ($\sigma_\nu \neq 0$) alone. **b**, Mean accuracy with AdaBS over 25 inference runs, the error bars corresponding to one standard deviation. AdaBS can almost fully compensate for the dependence of the drift exponent on the target conductance, and only a slight drop of $\sim 0.2\%$ over 1 year is observed when introducing random drift variability.

Supplementary Figure 6: Effect of random conductance variations on drift compensation methods

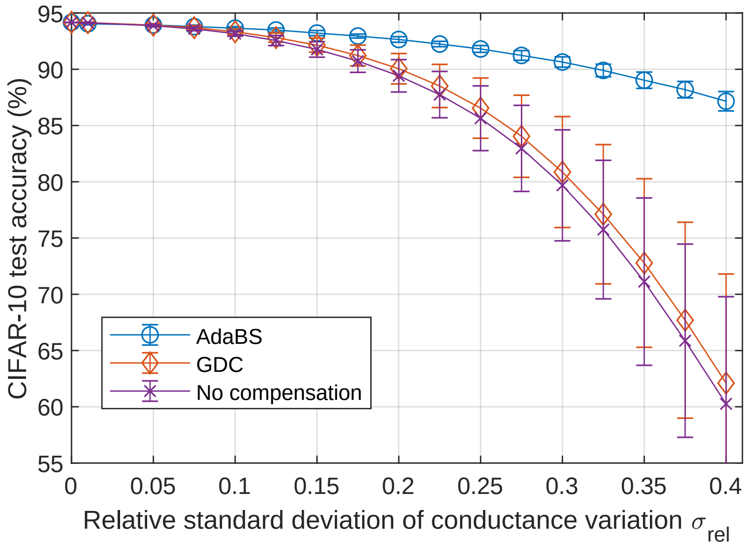

Supplementary Figure 6. **Effect of random conductance variations on drift compensation methods.** Test accuracy of ResNet-32 on CIFAR-10 as a function of relative standard deviation of conductance variations with AdaBS, GDC, and no compensation method. The aim of this simulation is to define a clear criterion as to how much random conductance variations can be mitigated by the drift compensation methods to be able to recover satisfactory network accuracy. We use a generic model for random conductance variations from the ideal target synaptic conductance $G^l_{\mathrm{T},ij}$ as $G^l_{ij} = G^l_{\mathrm{T},ij} \times \mathcal{N}(1, \sigma^2_{\mathrm{rel}})$. σ_{rel} is thus defined as the relative standard deviation of the actual conductance G^l_{ij} with respect to the ideal target conductance $G^l_{\mathrm{T},ij}$. Using such a generic model allows us to define a universal criterion, independent of the device technology. Moreover, this model approximates fairly well the random conductance variations due to drift variability and $1/f$ noise in PCM, where the magnitude of the variations is proportional to the programmed conductance value (see Supplementary Equations (16) and (17) of Supplementary Note 2). We performed inference simulations with ResNet-32 on CIFAR-10 using this model of device conductance to represent the weights. We employed GDC, AdaBS, and no compensation during inference with different values of σ_{rel}. The network was trained by injecting noise with $\eta_{\mathrm{tr}} = 3.8\%$. The results clearly show that AdaBS compensates much better for random conductance variations compared with GDC, which performs almost the same as when no compensation technique is used. We define the criterion as the maximum relative conductance variation σ_{rel} that can be tolerated to obtain an accuracy higher than 90%. By this definition, GDC can tolerate up to 20% variations, whereas for AdaBS it is 32.5%, an improvement of 1.6×. In terms of PCM drift variability, this corresponds approximately to a tolerable drift exponent standard deviation σ_ν of up to 0.013 for GDC, compared with 0.02 for AdaBS, when considering a time span of 6 orders of magnitude from programming (e.g. from 30 seconds to 1 year). The error bars represent the standard deviation over 25 inference runs.

Supplementary Figure 7: Effect of stuck devices on inference accuracy with additive noise training

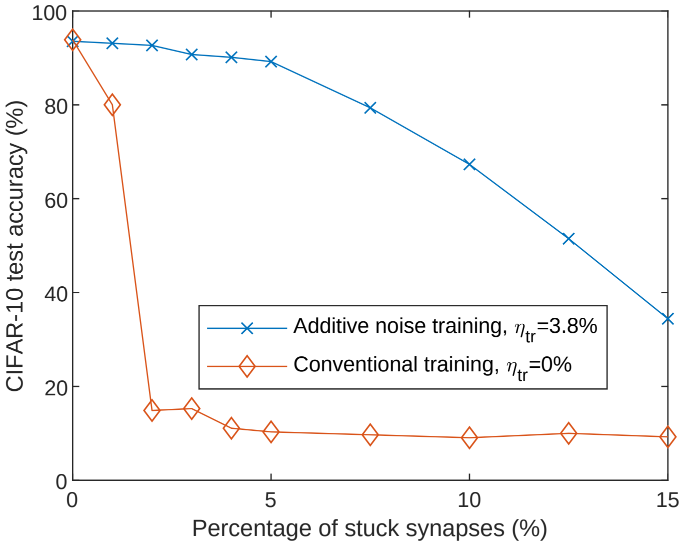

Supplementary Figure 7. **Effect of stuck devices on inference accuracy with additive noise training.** Test accuracy of ResNet-32 on CIFAR-10 as a function of stuck synapses during inference. We compare the accuracy obtained with weights trained using either the additive noise training technique (with $\eta_{tr} = 3.8\%$) or conventional training (e.g. no noise). For simulating the stuck device behavior during inference, we randomly selected a fraction (denoted as percentage of stuck synapses) of synapses for each layer. Each stuck synapse had its weight set to 0, $-W_{max}$ or W_{max} (W_{max} is the maximum absolute weight value of a layer). Note that the additive noise training algorithm that we employed did not take into account any of the stuck devices during training; the stuck devices were introduced only during inference. The results show that additive noise training is more robust to stuck faults compared to conventional training. With conventional training, the accuracy drops to 80% with just 1% of the devices stuck. On the other hand, using additive noise training allows the accuracy to remain above 90% for up to 4% stuck devices. This shows that additive noise training is effective in making the network robust to weight perturbations during inference, even for perturbations that are quite different from the generic noise applied during training.

Supplementary Figure 8: Effect of data converter quantization on accuracy

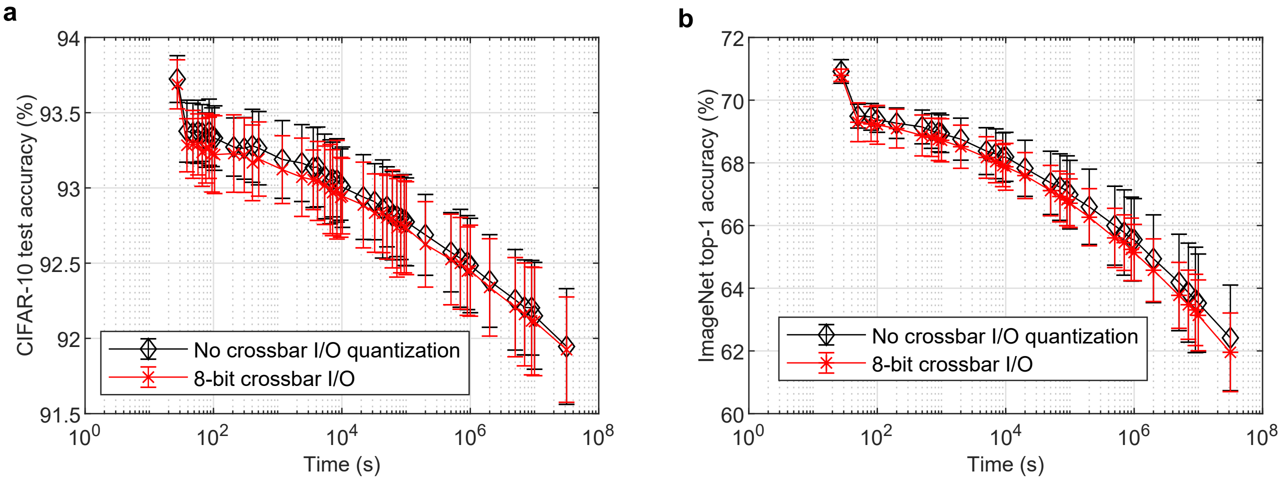

Supplementary Figure 8. **Effect of data converter quantization on accuracy.** Effect of 8-bit digital-to-analog/analog-to-digital conversions of input/output (I/O) of crossbar arrays during inference simulated using the PCM model with GDC. I/O quantization ranges are set to the 99.995-th percentile of the activation/preactivation distributions that are obtained when forward propagating 10k randomly sampled images from the training dataset through the baseline network. The I/O quantization ranges are computed only once and are kept constant during inference. The networks are trained with additive noise of $\eta_{\rm tr} = 3.8\%$, but not retrained with 8-bit quantization of activations/preactivations. The error bars correspond to the standard deviation over 25 inference runs. **a**, Effect of I/O quantization to 8-bit on the test accuracy of ResNet-32 on CIFAR-10. **b**, Effect of I/O quantization to 8-bit on the top-1 accuracy of ResNet-34 on ImageNet.

Supplementary Figure 9: PCM-based deep learning inference simulator

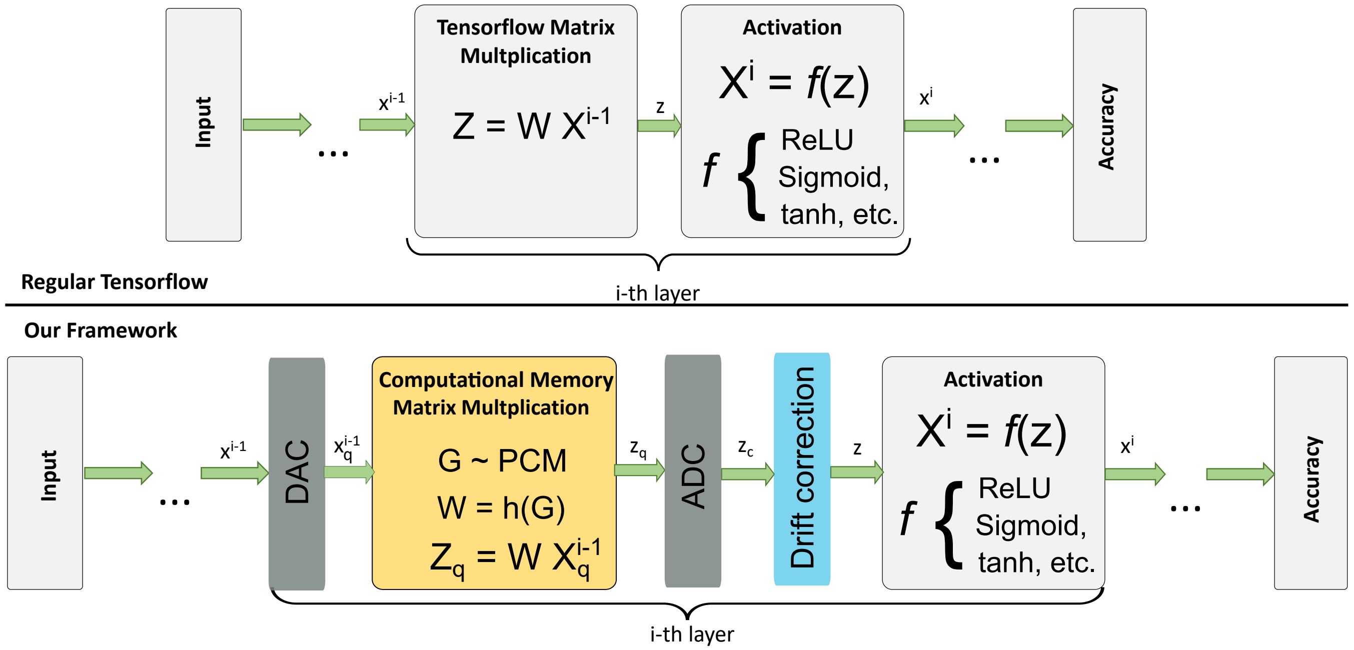

Supplementary Figure 9. **PCM-based deep learning inference simulator.** Schematic representation of the implementation of a fully-connected layer in our TensorFlow simulation framework. The regular TensorFlow matrix multiplication operation is replaced with a custom operation which takes into account the device model presented in Supplementary Note 2. Each custom matrix multiplication is also associated with configurable quantization of crossbar input/output to simulate digital-to-analog (DAC) and analog-to-digital (ADC) conversions. The drift correction module implements the drift correction techniques GDC (Supplementary Figure 3) and AdaBS (Supplementary Note 3). A similar structure holds for the convolution layers once the convolution kernels are re-arranged to form a matrix as described in Figure 1**b** of the main manuscript.

SUPPLEMENTARY NOTES

Supplementary Note 1: Fast initial convergence on ImageNet

Before starting the additive noise training of the ResNet-34 on the ImageNet dataset with $\eta_{\mathrm{tr}} = 3.8\%$, we performed an additive noise inference with $\eta_{\mathrm{inf}} = 3.8\%$ on the pretrained FP32 weights and achieved a top-1 accuracy of only 1.2%. This accuracy is very low and shows *a priori* that it is very hard to train such a network with additive noise. However, repeating the inference on the ResNet-34 network after only a 100 mini-batches of additive noise training leads already to a top-1 test accuracy of 65.9% as shown in Supplementary Figure 10.

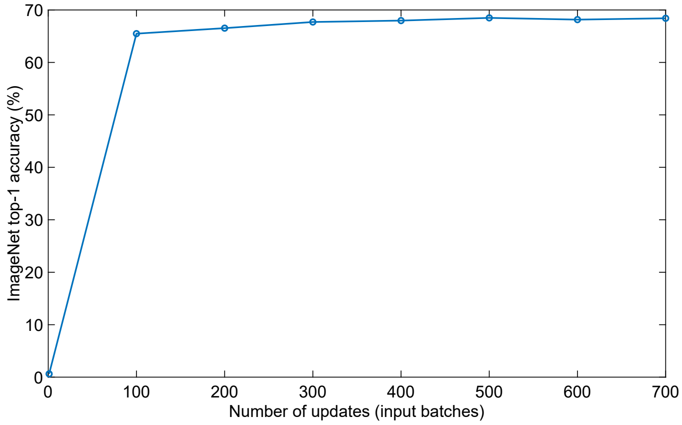

Supplementary Figure 10. Top-1 accuracy evolution of ResNet-34 on ImageNet as a function of training mini-batch during additive noise training with $\eta_{\mathrm{tr}} = \eta_{\mathrm{inf}} = 3.8\%$.

The main reason for this quick recovery in accuracy is the updating of the batch normalization statistics (μ and σ^2). The estimates of the statistics computed during the training phase become very inaccurate as soon as additive noise is injected in the network. Just by performing batch normalization statistics updates for 100 mini-batches without any other weight update, we are able to recover up to already 48.28% top-1 accuracy on the test dataset. This proves the aforementioned point and the results are shown in Supplementary Figure 11.

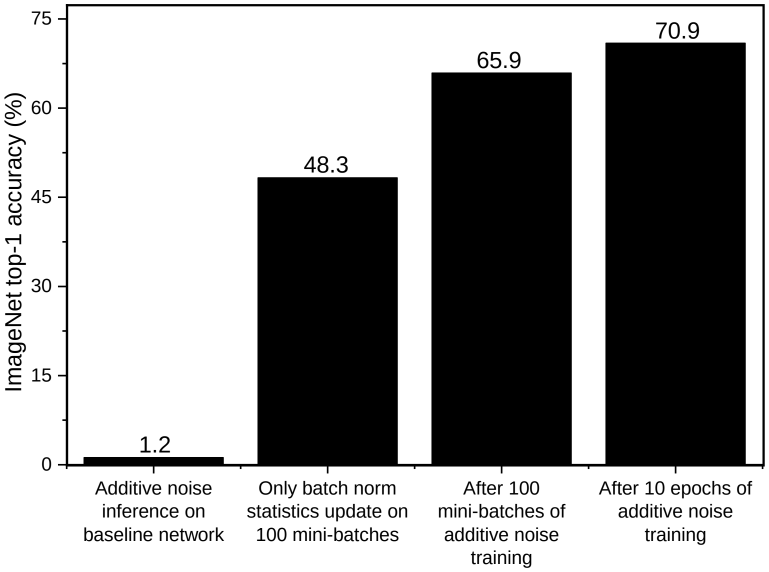

Supplementary Figure 11. Accuracy improvement with only batch normalization statistics updates for 100 mini-batches, 100 mini-batches of additive noise training, and 10 epochs of additive noise training. $\eta_{\mathrm{tr}} = \eta_{\mathrm{inf}} = 3.8\%$ is used.

We also recorded the evolution of the parameters of ResNet-34 during the first 100 training updates (parameter update by backpropagation and batch normalization statistics update). We computed the evolution of the L^2-norm of the difference between

the parameters and their initial value. Supplementary Figure 12 shows the L^2-norm values, confirming that the parameters changing the most during additive noise training are the weights of the first and last layers, the batch normalization parameters of the first layer and of the layers at the beginning of each ResNet block. It also suggests that the additional 17.62% gain in top-1 accuracy during the first 100 training updates that does not come from the batch normalization statistics update is mainly due to the weight updates from the first and last layers.

The findings described in this note are in agreement with the results shown in Supplementary Ref. 3.

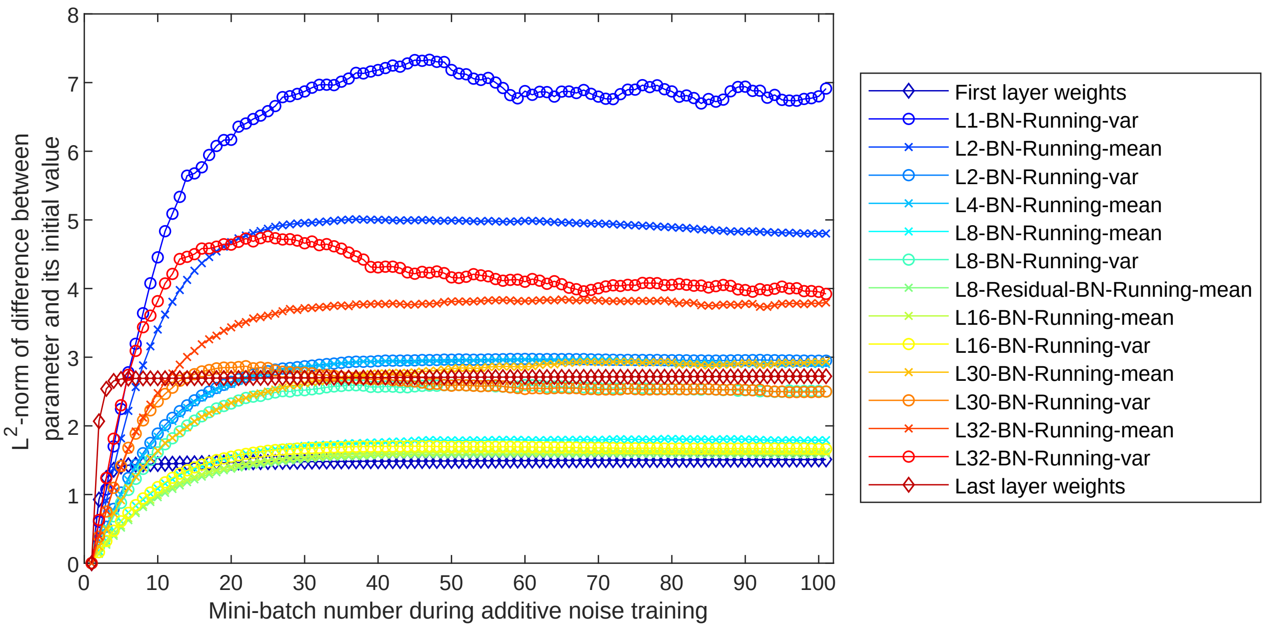

Supplementary Figure 12. Evolution of the L^2-norm of the difference between the network parameters and their initial value during additive noise training with $\eta_{tr} = 3.8\%$. Only the parameters for which the L^2-norm becomes larger than a threshold of 1.5 are shown.

Supplementary Note 2: Phase-change memory model for inference

Conductance drift

The conductance measured from the PCM is observed to drift over time according to the relation,

$$G(t) = G(t_0)(t/t_0)^{-\nu}, \tag{1}$$

where $G(t_0)$ is the conductance measured at time t_0 and ν is the drift coefficient[4]. The drift is attributed to the structural relaxation of an amorphous volume created after each programming event.

For a reliable estimate for the drift coefficient, we iteratively programmed 10,000 PCM devices to target conductance values, G_T, approximately at 23 μs and measured their temporal evolution until 10^5s, a time span of approximately 10 orders in magnitude. We obtained the drift coefficients of conductance evolution by fitting them to Supplementary Equation (1). The mean and standard deviation of the extracted drift coefficients are plotted as a function of the target conductance at 23 μs in Supplementary Figure 13.

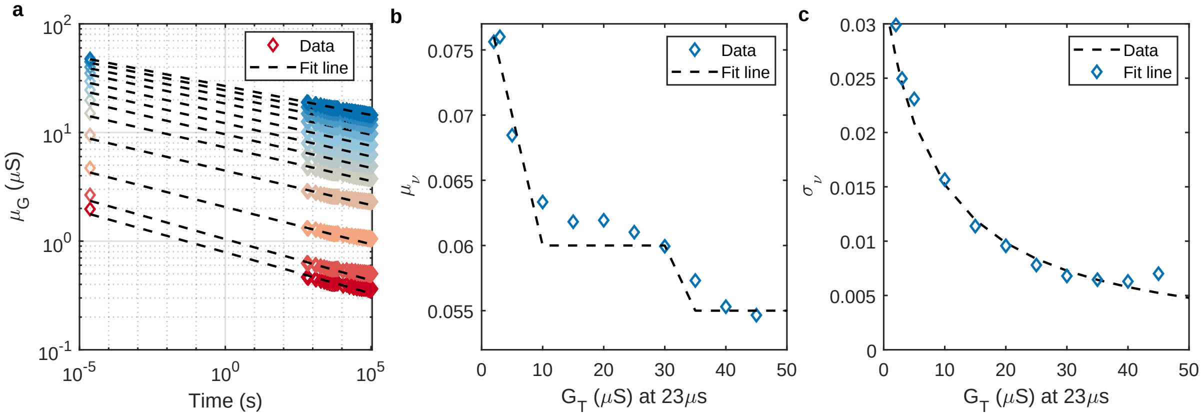

Supplementary Figure 13. **a,** Conductance drift observed in devices iteratively programmed to chosen target values at 23 μs. Corresponding fit lines based on Supplementary Equation (1) are also shown. **b,** Mean (μ_ν) and **c,** standard deviation (σ_ν) of the extracted drift coefficients, ν. Corresponding fit lines based on Supplementary Equation (2) and (3) are also shown.

We model ν as a Gaussian random variable with a mean, μ_ν, and standard deviation, σ_ν, which are functions of the target conductance values. The modeled equations for μ_ν and σ_ν are given below:

$$\mu_\nu = \max(-0.002G_\mathrm{T} + 0.08, \min(0.06, \max(-0.001G_\mathrm{T} + 0.09, 0.055))) \tag{2}$$
$$\sigma_\nu = 1/(3.5787G_\mathrm{T} + 30.0320) \tag{3}$$

Read noise

PCM is known to exhibit $1/f^\gamma$ noise[5]. The power spectral density, S_G, of the colored noise is given by,

$$\frac{S_G}{G^2} = \frac{Q}{f^\gamma}, \tag{4}$$

where G is the device conductance and Q is a factor determined by the phase configuration within PCM. The variance of the read noise, σ_nG^2, can be estimated by integrating S_G over the frequency range of measurement. For $\gamma \neq 1$,

$$\sigma_\mathrm{nG} = \frac{Q_\mathrm{s} G}{\sqrt{1-\gamma}} \sqrt{f_\mathrm{max}^{1-\gamma} - f_\mathrm{min}^{1-\gamma}} \tag{5}$$

where $Q_\mathrm{s} = \sqrt{Q}$. The f_max is determined by read pulse duration ($T_\mathrm{read} = 250\,ns$) in our experimental platform and f_min is determined by the time over which the noise is integrated. To estimate read noise variance from the device, the 10,000 devices, iteratively programmed at 23 μs, were read 50 times within a time window of $T = 90$ s, which was further repeated until 10^5s.

The fifty reads over the interval T were used to determine the read noise standard deviation as a function of time and conductance. $f_{\max} = 1/2T_{\text{read}}$ and $f_{\min} = 1/(T + T_{\text{read}})$. The average value of γ for the conductance range of operation was approximated to be 1.21 from an independent measurement. Q_s can be estimated from this information as a function of target conductance (Supplementary Figure 14**a**) and time (Supplementary Figure 14**b**). The behavior is captured using the relation,

$$Q_s(G_T, t) = K/G_T^{\alpha} \times (t/t_1)^{-\nu_q} \tag{6}$$

where $K = 0.0710$, $\alpha = 0.618$ and $t_1 = 1.089 \times 10^5$s. ν_q is observed to be dependent on the target conductance as shown in Supplementary Figure 14**c**. The fit line for ν_q is given by,

$$\nu_q = \min(0.081, 0.0150\,G_T + 0.02) - \min(0.035, -0.30 + 0.01\,G_T).(x > 30) \tag{7}$$

The match between the estimated and modeled read noise for different target conductance values is shown in Supplementary Figure 14**d**. While the read noise data points in Supplementary Figure 14**d** are based on noise integrated in a fixed time window of 90 s, for inference experiments we are interested in the read noise with respect to the initial programmed conductance values. Hence, read noise needs to be integrated from the point of iterative programming to the time instance at which we are performing the inference.[6] The read noise predicted by the model based on this growing time window is also shown in Supplementary Figure 14**d**.

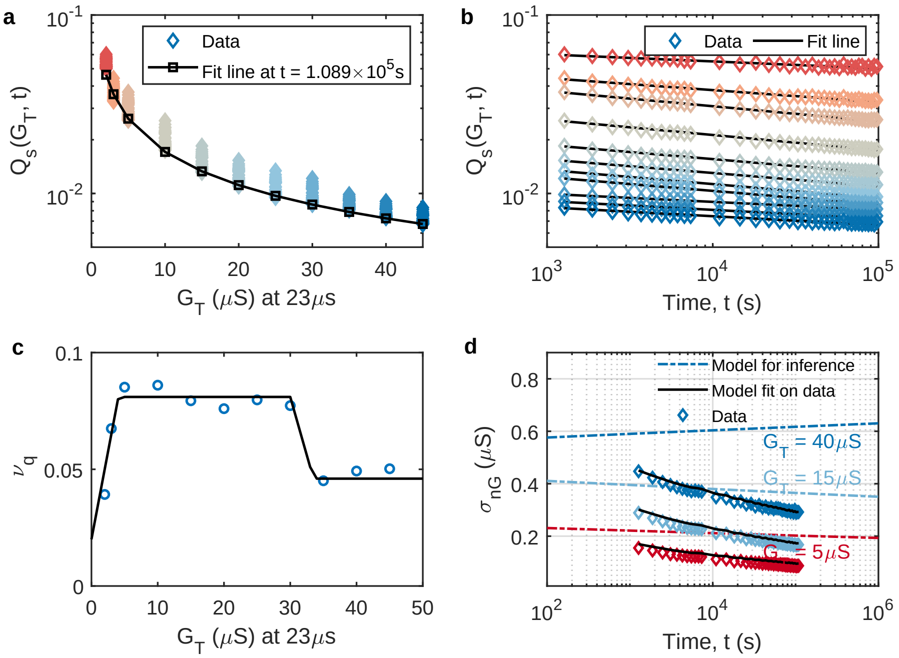

Supplementary Figure 14. Q_s as a function of target conductance G_T (**a**) and time (**b**). **c,** Estimated ν_q and model fit. **d,** Standard deviation of read noise σ_{nG} estimated from measured conductance samples over a 90 s window and corresponding model response. In the read noise prediction for inference, the time window of integration keep increasing from the point of iterative programming.

Mapping the model parameters from 23μs to 20 s

Inference experiments in the main article were performed based on the target conductance values defined at approximately 20 s. Since, the drift coefficients and read noise parameters are expected to be independent of conductance drift, we mapped the model Supplementary Equations (2, 3, 6, 7) to target conductance values defined at 20 s using the drift model. The modified equations in terms of the target conductance defined at 20s are shown below (also in Supplementary Figure 15).

$$\mu_\nu = \max(-0.0045G_T + 0.08, \min(0.06, \max(-0.00169G_T + 0.0825, 0.055))) \tag{8}$$

$$\sigma_\nu = 1/(8G_T + 30.0320) \tag{9}$$

$$Q_s(G_T, t) = 0.0388/G_T^{0.5729} \times (t/t_1)^{-\nu_q} \tag{10}$$

$$\nu_q = \min(0.081, 0.04\,G_T + 0.02) - \min(0.035, -0.2048 + 0.0155\,G_T).(x > 13.21) \tag{11}$$

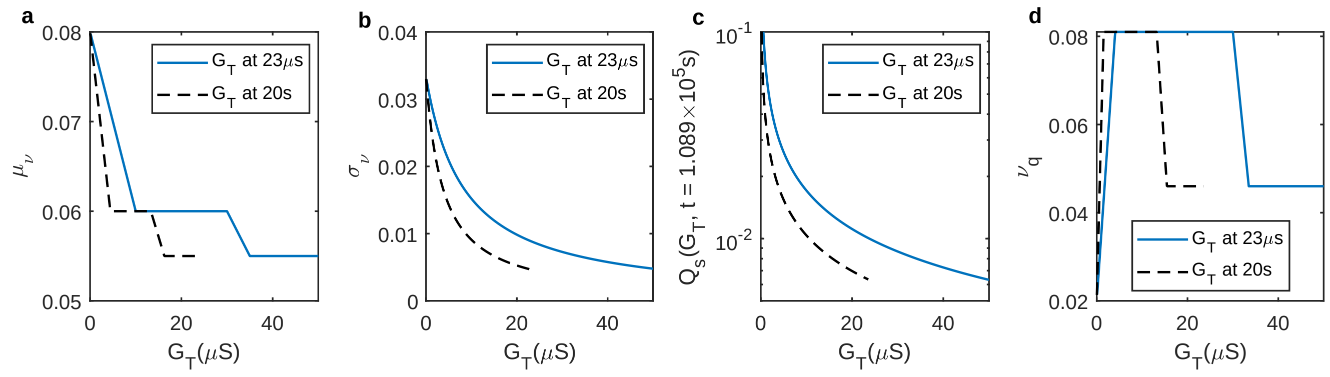

Supplementary Figure 15. The model parameters.

Programming noise

The conductance drift and slow evolving $1/f^{\gamma}$ noise makes accurately determining the programming noise of the PCM quite complex. Hence, the programming noise was determined so as to compensate for the difference between the variability predicted by the read noise and drift model and those exhibited by the device. We implemented two incremental programming noises in a state-dependent manner at two initial time points (T_0, T_1). The standard deviation of the programming noises as a function of target conductance values is shown in Supplementary Figure 16. The noise applied at T_0 is the experimentally measured programming noise at 27.36 s reported in Figure 3**b** of the main manuscript. The noise applied at T_1 captures additional short-term relaxation effects occurring after iterative programming[7]. The succeeding temporal evolution of the device was predicted using drift and read noise model (Supplementary Equations (8, 9, 10, 11)).

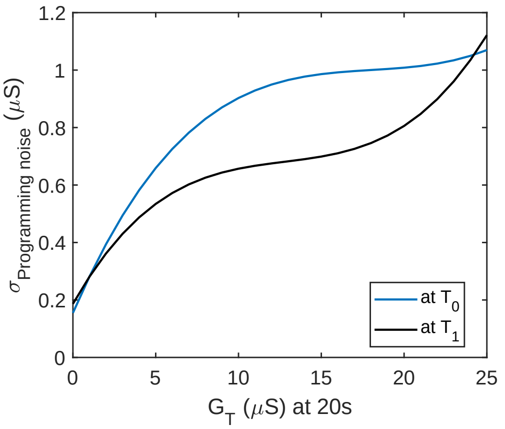

Supplementary Figure 16. Standard deviation of the programming noise added to the model at times $T_0 = 27.36\,\text{s}$ and $T_1 = 55.39\,\text{s}$.

Model response

Using the model equations, PCM conductance evolution as a function of time was simulated as follows:

$$\nu = \mathcal{N}(\mu_\nu(G_\mathrm{T}), \sigma_\nu(G_\mathrm{T})) \tag{12}$$

$$G(T_0) = G_\mathrm{T}(T_0/20)^{-\nu} + \mathcal{N}(0, \sigma_{\text{prog. noise}} \text{ at } T_0) \tag{13}$$

$$G(T_1) = G(T_0)(T_1/T_0)^{-\nu} + \mathcal{N}(0, \sigma_{\text{prog. noise}} \text{ at } T_1) \tag{14}$$

(15)

for all $t \geq T_1$

$$G_{\text{drift}}(t) = G(T_1)(t/T_1)^{-\nu} \tag{16}$$

$$\sigma_{\text{nG}} = \frac{Q_{\text{s}}(G_{\text{T}},t)G_{drift}(t)}{\sqrt{1-\gamma}}\sqrt{f_{\text{max}}^{1-\gamma} - f_{\text{min}}^{1-\gamma}} \tag{17}$$

where $f_{\text{min}} = 1/(t+T_{\text{read}})$. For all $t \geq T_1$, the modeled conductance, $G(t) = G_{\text{drift}}(t) + \mathscr{N}(0, \sigma_{\text{nG}})$.

Supplementary Note 3: Adaptative batch normalization statistics (AdaBS) update technique

Drift in a PCM device has a dependence on the initial target conductance value of the device and varies across different devices in an array. As a result, the decrease in conductance values occurs at different rates across devices. The read noise of the PCM devices also depends on the initial target conductance, the actual value of the PCM conductance, and has a $\frac{1}{f^{\gamma}}$ frequency dependence. All these effects corrupt the weight distribution and hence the activation distribution of a DNN layer, causing the accuracy to degrade over time. The batch normalization layer can be leveraged to correct the activation distribution out of a PCM crossbar array, as it can adapt the optimal statistics (i.e. mean and variance) of its inputs (i.e. outputs of a crossbar) required for normalization. We demonstrate an advanced drift correction technique **ada**ptive **b**atch normalization **s**tatistics (AdaBS) that can improve the accuracy retention over time and outperforms the global drift compensation (GDC) method shown in Supplementary Figure 4.

Methodology

Supplementary Figure 17 shows the batch normalization layer operation both in training and inference phases, along with the added AdaBS calibration. The batch normalization layer has different behavior in training and inference phase of a DNN. During the training of a DNN, a batch normalization layer normalizes its input to zero mean and unit variance by computing the mean (μ_{B}) and variance (σ_{B}^2) over a batch of images. The normalized input is then scaled and shifted by γ and β. During the training phase, γ and β are learned through backpropagation. In parallel, a global running mean (μ) and variance (σ^2) are computed by exponentially averaging μ_{B} and σ_{B}^2 respectively, over all the training batches. After training, the estimates of the global mean and variance μ and σ^2 are then used during the inference phase. When performing forward propagation during inference, the batch normalization coefficients μ, σ^2, γ, and β are used for normalization, scale, and shift without computing mini-batch mean (μ_{B}) and variance (σ_{B}^2).

The calibration phase of AdaBS consists in recomputing and updating μ and σ^2 for every layer where batch normalization is present. We recompute μ and σ^2 by feeding a randomly sampled set of mini-batches from the training dataset. In recomputing μ and σ^2, hyper-parameters such as mini-batch size (m) and momentum (p) need to be carefully tuned to achieve the best network accuracy.

For AdaBS calibration, we observed that using an optimal value of the momentum is necessary to achieve good inference accuracy evolution over time. For this, we have developed an algorithm to estimate the optimal value of momentum by an empirical analysis. Consider the exponential accumulation of any statistics S of a batch normalization layer as given by Supplementary Equation (18). Too high momentum value will result in more contribution from the initial statistic S_0 and lower contribution from the statistics computed over the n mini-batches used to recalibrate the final statistic S. On the other hand, with a too low

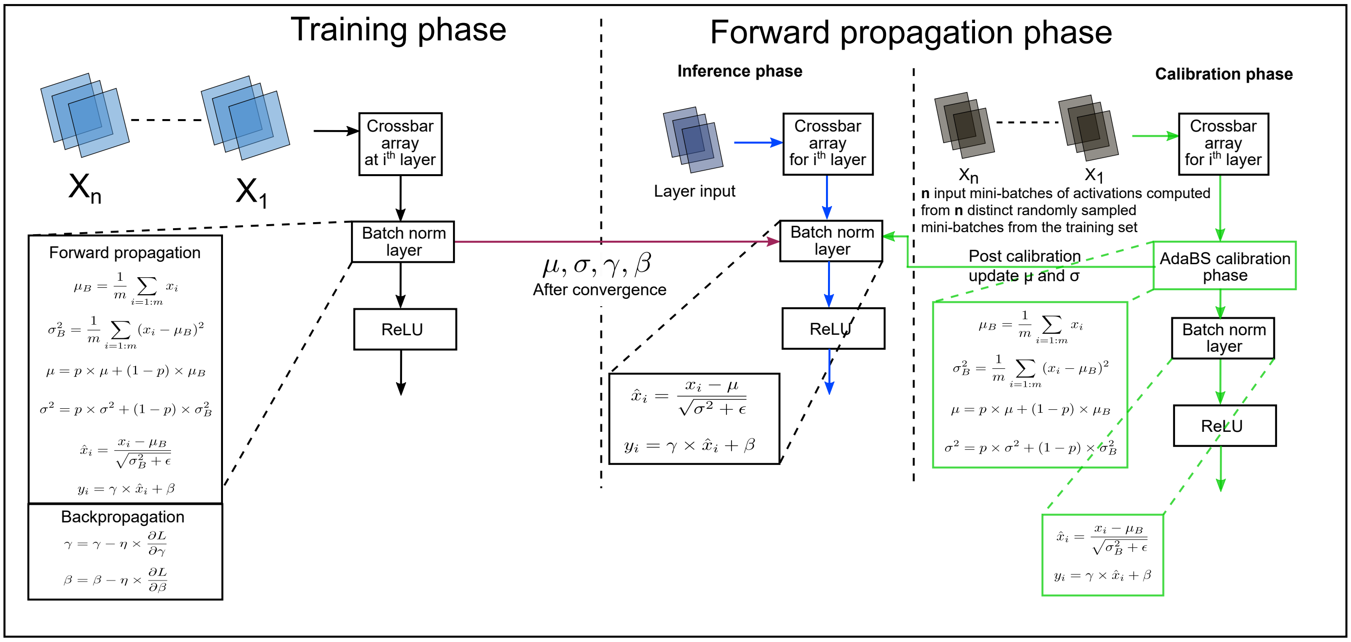

Supplementary Figure 17. Batch normalization operations during training, inference, and AdaBS calibration.

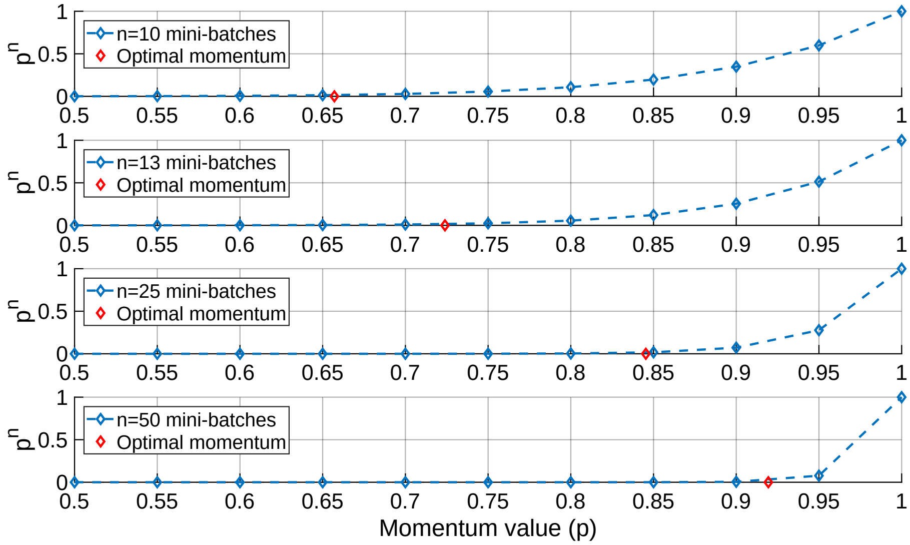

Supplementary Figure 18. Estimating optimal momentum value (p) for AdaBS implementation for a required number of mini-batch statistic updates (n) in a batch normalization layer. The optimal momentum is the value of the momentum at which the quantity p^n is 0.015. The value of 0.015 was found empirically to give the best overall network performance with AdaBS.

momentum, the final statistic will mostly be computed from the last injected training mini-batch. In practice, this results in the fact that the proportion of the initial statistic S_0 in the resulting average S, prop_{S_0}, has to be under a threshold t, and that the n injected batches must be represented in equal proportions in the final statistic S. In practice, a value of $t = 0.015$ fulfills the above mentioned conditions (see Supplementary Figure 18). Supplementary Equations (18), (19), and (20) show the derivation in detail when injecting n mini-batches to update the batch normalization statistics.

$$S = p^n \times S_0 + (1-p) \times \sum_{i=1:n} S_i \times p^{n-i} \tag{18}$$

$$\text{prop}_{S_0} = \frac{p^n}{p^n + (1-p) \times \sum_{i=1:n} p^{n-i}} = p^n \tag{19}$$

$$p = 0.015^{(1/n)} \tag{20}$$

To find the optimal number of calibration images as a fraction of the training dataset of the ResNet-32 network, we fixed the mini-batch size to $m = 200$ images. We performed AdaBS calibration on the ResNet-32 network with the weights read from the PCM hardware inference experiment. Supplementary Figure 19 shows the accuracy evolution with AdaBS for different fractions of the training dataset, with the optimal momentum computed using Supplementary Equation (20). From these results, we observe that 5% of the training set is sufficient for the AdaBS calibration of ResNet-32 network. The accuracy retention with 5% of the training set is comparable to that obtained when using the entire training set.

Computational and memory overhead of AdaBS

Better accuracy retention is obtained at the cost of additional digital operations AdaBS requires during the calibration phase. Supplementary Figure 20 shows the amount of digital computations required in the calibration and inference phases. The forward propagation operations are the number of operations all the batch normalization layers require for 10k CIFAR-10 images in ResNet-32. Calibration phase operations are the computations required to calibrate the coefficients of all the batch normalization layers in ResNet-32. In the forward propagation, both GDC and AdaBS do not incur any additional computations. However, during calibration phase, GDC uses fewer computations for computing calibration coefficients compared with AdaBS. The main intuition behind the GDC method is to compute a measure of change in the spread of the conductance values in

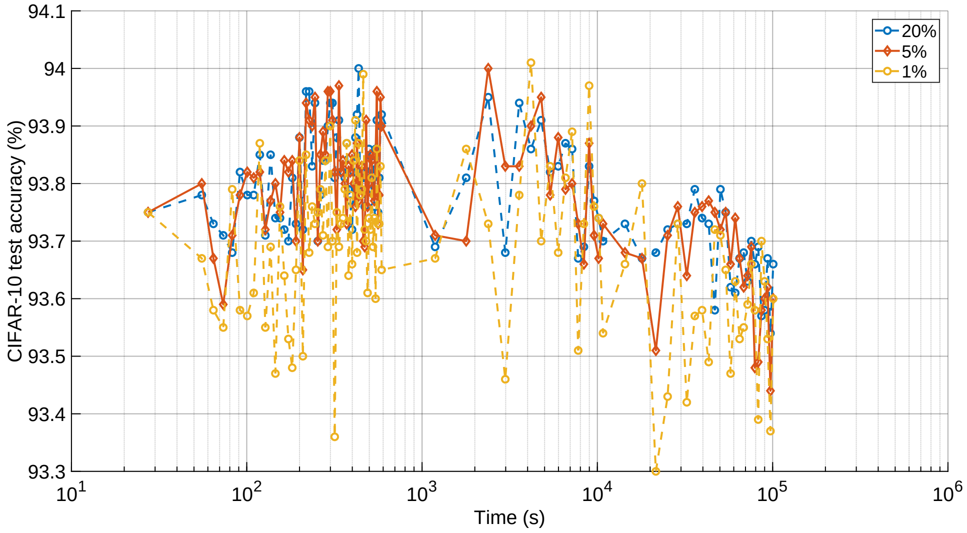

Supplementary Figure 19. Empirical study to obtain an optimal number of images as a fraction of training dataset for the AdaBS calibration. AdaBS is implemented on the ResNet-32 network with the weights read from the PCM hardware inference experiment using a mini-batch size of $m = 200$.

Method	Forward propagation on 10K images		Calibration phase	
	Multiplications	Additions	Multiplications	Additions
No drift correction	$2.98\text{x}10^9$	$2.98\text{x}10^9$	0	0
GDC	$2.98\text{x}10^9$	$2.98\text{x}10^9$	34	1110
AdaBS (L2-BN)	$2.98\text{x}10^9$	$2.98\text{x}10^9$	$7.76\text{x}10^8$	$2.33\text{x}10^9$
AdaBS (L1-BN)	$2.98\text{x}10^9$	$2.98\text{x}10^9$	$8.58\text{x}10^4$	$2.33\text{x}10^9$

Supplementary Figure 20. Analysis of computational overhead of AdaBS compared with regular batch normalization implementation for inference of the ResNet-32 network trained on CIFAR-10 dataset. Forward propagation operations are the total number of multiplications and additions required for all batch normalization layers with 10K CIFAR-10 images (test dataset). Calibration phase operations are the total number of multiplications and additions required for calibrating batch normalization layer coefficients with AdaBS, or for computing the global scaling factor $\hat{\alpha}$ for GDC. The AdaBS calibration is performed with $m = 200$ and $n = 13$ (2,600 images).

a crossbar array by estimating ratio of 1-norm of conductance matrix at two distinct time instances. Due to this, the GDC calibration is independent of the input dataset used by the network. As a result, GDC has a smaller computational overhead during the calibration phase compared to AdaBS. AdaBS requires more computations during calibration as it computes the first and second order moments of the crossbar outputs over a set of images.

A batch normalization layer can be implemented with different statistical estimation for center and spread instead of mean and variance with small impact on the network accuracy[8]. In our computation overhead estimates, we therefore considered two versions of batch normalization as follows:

- L2-BN: Original batch normalization layer with mean and variance as statistical estimation of center and spread, respectively, for the batch normalization layer inputs.
- L1-BN: A batch normalization layer with mean and mean absolute deviation as statistical estimation of center and spread, respectively, for the batch normalization layer inputs.

Out of these two implementations, L1-BN requires fewer multiplication operations to compute the estimation of spread, i.e. absolute mean deviation, compared to that of L2-BN. We show in Supplementary Figure 21 that with L1-BN implementation the ResNet-32 network accuracy and its evolution are not affected compared to L2-BN implementation.

Besides the computational overhead, AdaBS requires additional memory resources to store the calibration images. For CIFAR-10, it needs $32 \times 32 \times 3 \times 2600 = 8$ MB, and for ImageNet $224 \times 224 \times 3 \times 1300 = 196$ MB. Clearly, this amount of data is too large to be stored in on-chip memory. Hence, it would have to be stored either in off-chip DRAM or non-volatile memory (Flash, PCM, RRAM, etc.). A simple analysis of the overhead from the off-chip data transfers can be made assuming off chip links with energy consumption close to 1pJ/bit[9,10]. This results in an energy per image equal to 24.6 nJ for CIFAR-10 and 1.2 μJ for ImageNet. For the overall sizes of the calibration sets for the two datasets, we obtain 64 μJ/calibration for CIFAR-10 and 1.3 mJ/calibration for ImageNet. Further, using 13Gbps and a conservative estimation of a realistic bandwidth[9,10], we obtain

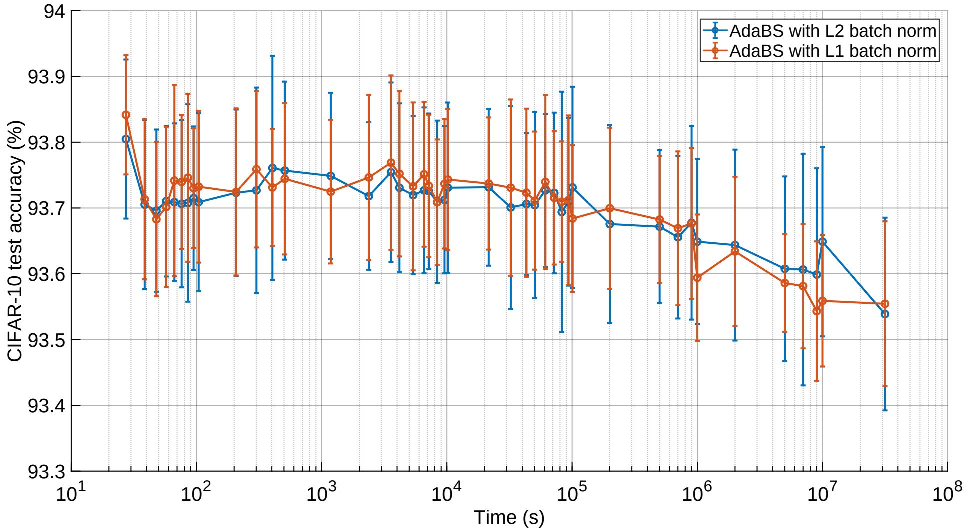

Supplementary Figure 21. Comparison of accuracy evolution with AdaBS between L1-BN and L2-BN with ResNet-32 on CIFAR-10 simulated using the PCM model. The networks were trained with additive noise ($\eta_{\mathrm{tr}} = 3.8\%$) and the respective batch normalization implementation (L^1-norm or L^2-norm). The error bars correspond to the standard deviation over 25 inference runs.

a transfer time equal respectively to 3.2 ms and 78.3 ms for CIFAR-10 and ImageNet. This overhead is reasonable given that the calibration is expected to be performed at time intervals larger than 10 seconds. Moreover, it can be performed less frequently as time elapses because the conductance changes due to drift are a logarithmic function of time. Finally, note that the AdaBS calibration could be potentially done as well using the images seen by the device during inference, which would avoid resorting on pre-stored calibration images.

Supplementary Note 4: Comparison with other noise injection methods for training

To compare the effectiveness of the additive noise injection technique proposed in this manuscript with other noise injection methods, we considered the four noise injection methods described below. We used the same training procedure and hyperparameters as for the experiments presented in the main manuscript, unless specified otherwise.

Noise injection on the input activations

We studied noise injection by inputting noise on the input activations of every layer during training. As shown in Supplementary Equation (21), for any arbitrary layer with weight matrix W, noise is added to the input activations X of the layer, leading to a noisy output Y. The noise is Gaussian distributed with zero mean and variance σ_{in}^2.

$$Y = W \times (X + \mathcal{N}(0, \sigma_{\text{in}}^2)) \tag{21}$$

We defined the ratio of the standard deviation of additive noise injected on activations (σ_{in}) to the spread of the input activation ($c \times \sigma_X$) to be the same as η_{tr} as given by Supplementary Equation (22).

$$\frac{\sigma_{\text{in}}}{c \times \sigma_X} = \eta_{\text{tr}} \tag{22}$$

It follows that

$$\sigma_{\text{in}} = c \times \sigma_X \times \eta_{\text{tr}}, \tag{23}$$

where σ_X is the standard deviation of the input activations and c is tunable hyper-parameter for a given layer.

Since there is no evident prior value for c, adapting its value for each convolutional layer such that Y is equal in distribution both for the cases of additive noise training on weights and input activations, is a reasonable choice. For this, we performed a Kolmogorov Smirnov test to compare the distributions of Y between both methods, and chose the value of c that led to the smallest Kolmogorov Smirnov score. We experimentally found out that the value of c is the same for the layers within a ResNet-block. The values of c that gave the highest accuracy on ResNet-32 after transfer to PCM synapses with $\eta_{\text{tr}} = 3.8\%$, found in a completely ad-hoc manner, were $c = 4$ for the first convolution layer, $c = 4$ for block 1 and 2, $c = 2$ for block 3, and $c = 4$ for the last fully-connected layer. We also had to clip weights of the first convolution layer to the range $[-0.8 \times \sigma_W, 0.8 \times \sigma_W]$.

Noise injection on the preactivations

Noise injection on preactivations has been often proposed for training DNNs for deployment on analog mixed-signal hardware[3,11]. Noise is added to the preactivations of the layer as per Supplementary Equation (24), for any arbitrary layer with weight matrix W, inputs activations X and output Y. The noise is Gaussian distributed with zero mean and variance σ_{preact}^2.

$$Y = W \times X + \mathcal{N}(0, \sigma_{\text{preact}}^2) \tag{24}$$

Again, for consistency, we defined the ratio of standard deviation of additive noise injected on preactivations (σ_{preact}) to the spread of the preactivation ($d \times \sigma_X \times W_{\text{max}}$) to be the same as η_{tr} as given by Supplementary Equation (25).

$$\frac{\sigma_{\text{preact}}}{d \times \sigma_X \times W_{\text{max}}} = \eta_{\text{tr}} \tag{25}$$

It follows that

$$\sigma_{\text{preact}} = d \times \sigma_X \times W_{\text{max}} \times \eta_{\text{tr}}, \tag{26}$$

where σ_X is the standard deviation of the input activations, W_{max} is maximum absolute value of the weights of a given layer, and d is tunable hyper-parameter for a given layer.

The variance is computed by matching the distributions of Y between additive noise injection on weights and additive noise injection on the preactivations for different values of d using the Kolmogorov Smirnov test, as explained earlier for additive noise on input activations. The values of d that gave the highest accuracy on ResNet-32 after transfer to PCM synapses with $\eta_{\text{tr}} = 3.8\%$, found in a completely ad-hoc manner, were $d = 2.5$ for the first convolution layer, $d = 6$ for block 1, 2 and 3, and $d = 7$ for the last fully-connected layer.

Noise injection on the network training dataset

We investigated a simple noise injection technique of injecting additive noise on the training dataset. This method can be seen simply as data augmentation, but since the data noise will flow through the whole network, it could help make the network more robust to weight perturbations during inference. As shown in Supplementary Equation (27), for an input training image X_{image}, noise is added on every pixel of the image. The noise is Gaussian distributed with zero mean and variance σ_{data}^2. The noisy image $\hat{X}_{\text{image}}$ is used for training the network.

$$\hat{X}_{\text{image}} = X_{\text{image}} + \mathcal{N}(0, \sigma_{\text{data}}^2) \tag{27}$$

We defined the variance of the additive noise on input dataset to be related to η_{tr} as given in Supplementary Equation (28)

$$\sigma_{\text{data}} = g \times \eta_{\text{tr}}, \tag{28}$$

where g is tunable hyper-parameter. The optimally tuned value of g that gave the highest accuracy on ResNet-32 after transfer to PCM synapses with $\eta_{\text{tr}} = 3.8\%$ was $g = 0.3$. This value cannot be deduced from any noise characterized on the PCM hardware.

Multiplicative noise injection on the weights

We studied multiplicative noise injection on the weights by multiplying the weights of every layer with Gaussian distributed noise during training. As shown in Supplementary Equation (29), weights are multiplied by a Gaussian distributed noise term for any arbitrary layer with weight matrix W, input activations X, and output Y. The noise term has unity mean to preserve the mean value of the weights and variance σ_{prod}^2.

$$Y = (W \times \mathcal{N}(1, \sigma_{\text{prod}}^2)) \times X \tag{29}$$

Similar to additive weight noise, there is a range of values of σ_{prod} over which the network achieves close to baseline accuracy after training. Even if the range of σ_{prod} values that the multiplicative Gaussian noise can take is broad enough, it is hard to map the observed PCM noise to σ_{prod}. One could think of a linear fit of the observed standard deviations of Figure 3**b** of the main manuscript, and setting the bias of the fit to 0. In this case, however, the noise would be underestimated and result in sub-optimal accuracy. The optimal value of σ_{prod} that gave the highest accuracy on ResNet-32 after transfer to PCM synapses was 0.1.

Results

Supplementary Figure 22 summarizes the results obtained with the four aforementioned methods for ResNet-32 on CIFAR-10, compared with the additive noise on weights method proposed in the main manuscript. As seen in Supplementary Figure 22**a**, the training procedure with all four methods could be adjusted such that a similar accuracy after transfer to PCM synapses is obtained. However, as described above, all four methods require one or multiple noise scaling factor hyperparameters to tune in order to reach satisfactory accuracy after transfer to PCM synapses. The values of these hyperparameters cannot be deduced from hardware observations in a straightforward manner. Therefore, in practice, it would be required to test trained weights multiple times on hardware for different hyperparameter values in order to achieve satisfactory performance, which is undesirable. In contrast, the methodology proposed in the main manuscript, which uses additive noise on weights, enables to estimate the magnitude of the weight noise to inject during training η_{tr} from a simple one-time hardware characterization. Moreover, the exact value of η_{tr} does not have to be determined in a very precise manner, because there is a wide range of values that lead to similar accuracy after transfer to PCM synapses (see Supplementary Figure 3). Therefore, a single representative value of η_{tr} could be used for training a network and deploying it on multiple chips, as long as a similar device technology and programming algorithm are used, and chip-to-chip variations are not too significant. Finally, we also found that training methods based on weight noise achieve a better accuracy retention over time (see Supplementary Figure 22**b**), suggesting that weight noise mimics the behavior of the PCM hardware better.

a

Noise injection method	Accuracy after training	Accuracy after transfer to PCM	Accuracy after a day	Noise scaling factor hyperparameter tuning
Input dataset	94.16%	93.55% ± 0.15	92.50% ± 0.30	Single hyperparameter sweep
Layer inputs	94.05%	93.50% ± 0.19	92.34% ± 0.34	Multiple hyperparameters to sweep
Layer preactivations	94.07%	93.55% ± 0.19	92.50% ± 0.31	Multiple hyperparameters to sweep
Multiplicative noise on weights	94.05%	93.69% ± 0.12	92.81% ± 0.28	Single hyperparameter sweep
Additive noise on weights	94.17%	93.72% ± 0.15	92.77% ± 0.29	No hyperparameter (known from hardware characterization)

b

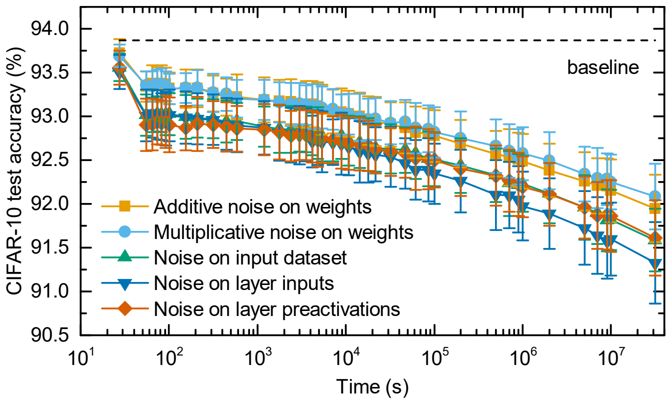

Supplementary Figure 22. **a**, Comparison between different methods of adding noise to the ResNet-32 network during training. The hyper-parameters for all methods were optimized as to give the highest accuracy on CIFAR-10 after transfer to PCM synapses, except for additive noise on weights where $\eta_{tr} = 3.8\%$, given by the hardware characterization, was used. **b**, Test accuracy over time of ResNet-32 on CIFAR-10 with GDC computed with the PCM model for the five different training methods. Methods based on weight noise achieve a better accuracy retention.

Supplementary Note 5: Energy efficiency and area of a computational PCM core

We calculated the energy efficiency of a 512×512 computational PCM core operating at a cycle time of 100 ns. We took into consideration the following contributions:

- Analog contributions:
  - Read Voltage Regulation: The energy for the voltage regulators used to keep the array columns at constant potential.
  - Analog Computation: The energy consumption of the computation being performed in the analog domain. We assume the average conductance of the PCM devices to be 2.5 μS and the read voltage to be 0.2 V.
  - ADCs: The energy for the analog-to-digital converters (ADCs) is obtained from Supplementary Ref. 12.
- Digital contributions:
  - PWM: The input data of the analog array is applied as voltage pulses by the pulse-width modulator (PWM). This contribution accounts for the energy of the modulator to perform a digital-to-analog conversion from an 8-bit signed number to a read voltage pulse.
  - Data Transfer (Input/Output): The energy of the data transfer operation of the input vector to the registers of the PWM modulator and to transfer the data produced by the ADCs.
  - Batch Normalization and ReLU: The energy of the digital processing element that applies Batch Normalization and ReLU nonlinearity to the pre-activations converted to the digital domain by the ADCs.
  - On-Chip Links: We take into consideration the energy of the core-to-core communication of the activations, including the energy of the digital logic that controls the links.
  - Digital Logic: The energy of the digital logic enforcing the dataflow. This logic takes care of storing the activations received from the core implementing the previous layer and fetching the input volume for the dot product to be executed at a given timestep.

The power consumption of the digital blocks was obtained from RTL simulations of the HDL code of a computational memory-based accelerator design. We estimated the overall energy efficiency to be 11.9 TOPS/W.

Regarding area occupation, we considered the ADCs to be the size of the ADCs presented in Supplementary Ref. 12. The number of ADCs used is 1/4 of the number of columns (so 4$\times$ multiplexing). The resulting area for the analog part of the array of size 512×512, including PWM and ADCs, was 0.42 mm^2. Further, from hardware simulations of the RTL code executing Batch Normalization and ReLU, we extrapolated its area to be in the ballpark of 0.15 mm^2. Overall, the area of a computational memory core was approximated to roughly 0.57 mm^2.

SUPPLEMENTARY REFERENCES

[1] He, K., Zhang, X., Ren, S. & Sun, J. Deep residual learning for image recognition. In *Proceedings of the IEEE conference on computer vision and pattern recognition*, 770–778 (2016).

[2] Le Gallo, M., Sebastian, A., Cherubini, G., Giefers, H. & Eleftheriou, E. Compressed sensing with approximate message passing using in-memory computing. *IEEE Transactions on Electron Devices* **65**, 4304–4312 (2018).

[3] Rekhi, A. S. *et al.* Analog/mixed-signal hardware error modeling for deep learning inference. In *Proceedings of the 56th Annual Design Automation Conference*, 81:1–81:6 (ACM, 2019).

[4] Ielmini, D., Sharma, D., Lavizzari, S. & Lacaita, A. Reliability impact of chalcogenide-structure relaxation in phase-change memory (PCM) cells, part I: Experimental study. *IEEE Trans. Electron Devices* **56**, 1070–1077 (2009).

[5] Nardone, M., Kozub, V., Karpov, I. & Karpov, V. Possible mechanisms for 1/f noise in chalcogenide glasses: A theoretical description. *Physical Review B* **79**, 165206 (2009).

[6] Nandakumar, S. R. *et al.* Phase-change memory models for deep learning training and inference. In *26th IEEE International Conference on Electronics, Circuits and Systems (ICECS)*, 727–730 (2019).

[7] Degraeve, R. *et al.* Quantitative model for post-program instabilities in filamentary RRAM. In *IEEE International Reliability Physics Symposium (IRPS)*, 6C–1–1–6C–1–7 (2016).

[8] Santurkar, S., Tsipras, D., Ilyas, A. & Madry, A. How does batch normalization help optimization? In *Advances in Neural Information Processing Systems*, 2483–2493 (2018).

[9] Tajalli, A. *et al.* A 1.02pJ/b 417Gb/s/mm USR link in 16nm FinFET. In *2019 Symposium on VLSI Circuits*, C92–C93 (2019).

[10] Zimmer, B. *et al.* A 0.11 pJ/Op, 0.32-128 TOPS, scalable multi-chip-module-based deep neural network accelerator with ground-reference signaling in 16nm. In *2019 Symposium on VLSI Circuits*, C300–C301 (2019).

[11] Klachko, M., Mahmoodi, M. R. & Strukov, D. Improving noise tolerance of mixed-signal neural networks. In *International Joint Conference on Neural Networks (IJCNN)*, 1–8 (2019).

[12] Kull, L. *et al.* 28.5 a 10b 1.5GS/s pipelined-SAR ADC with background second-stage common-mode regulation and offset calibration in 14nm CMOS FinFET. In *2017 IEEE International Solid-State Circuits Conference (ISSCC)*, 474–475.
